# Assessing proprioception in children with upper motor neuron lesions: feasibility, validity, and reliability of the proprioception measurement tool

**DOI:** 10.3389/fresc.2024.1373793

**Published:** 2024-08-09

**Authors:** Petra Marsico, Lea Meier, Anke Buchmann, Andrina Kläy, Marietta L. van der Linden, Thomas H. Mercer, Hubertus J. A. van Hedel

**Affiliations:** ^1^ Research Department, Swiss Children’s Rehab, University Children’s Hospital Zurich, Affoltern am Albis, Switzerland; ^2^ Children’s Research Center CRC, University Children’s Hospital Zurich, University of Zurich, Zurich, Switzerland; ^3^ Centre for Health, Activity and Rehabilitation Research, Queen Margaret University, Edinburgh, Scotland

**Keywords:** outcome measures, cerebral palsy, psychometrics, movement control, rehabilitation

## Abstract

**Introduction:**

To investigate the feasibility, discriminative and convergent validity, and reliability of a lower limb sensor-based proprioception measure in children with upper motor neuron (UMN) lesions.

**Method:**

We assessed three proprioception modalities (joint movement, joint position, and dynamic position sense) of the lower limbs in 49 children with UMN lesions and 50 typically developing (TD) peers (5–19 years). Forty-three children with UMN lesion had a congenital and six an acquired brain lesion and 82% were able to walk without a walking aid. We evaluated the feasibility, compared the test results between children with UMN lesions and TD peers, and calculated Spearman correlations (r_s_) between the modalities. We quantified relative reliability with Intra-Class Correlation Coefficients (ICC) and absolute reliability with Smallest Detectable Changes (SDC).

**Results:**

Most children with UMN lesions (>88%) found the tests easy to perform. The children with UMN lesions had significantly (*p* < 0.001) lower proprioceptive function than the TD children. The correlation between the three proprioceptive modalities was moderate to high (0.50 ≤ r_s_ ≤ 0.79). The relative reliability for test-retest and the inter-rater reliability was moderate to high (ICCs = 0.65–0.97), and SDC was between 2° and 15°.

**Discussion:**

The three tests are feasible, and discriminative and convergent validity and reliability were confirmed. Further studies should investigate the influence on motor function and performance in children with UMN lesions.

## 1 Introduction

Proprioception is a crucial internal input that involves the perception of the body's position and movement in space (1). It conveys information to the brain on the relative positions of body parts, crucial for movement coordination and balance (2). Proprioception comprises of different modalities such as joint movement sense (JMS) or kinaesthesia, which detects the onset and direction of joint movement, joint position sense (JPS or statesthesia) for detecting joint position, and dynamic position sense (DPS) for monitoring limb position during active movement (1).

In a recent Delphi study on somatosensory assessment of the lower limbs, experts agreed that three proprioceptive modalities, namely JMS, JPS, and DPS, are relevant to gait and balance in children with UMN lesions (3). Nevertheless, to date, we do not have the assessments to investigate these different modalities. Further, for the few assessments that exist, there is no evidence of the psychometric properties when applied in children with upper motor neuron (UMN) lesions (4).

The few available studies on proprioceptive assessments in this population investigated differences between children with UMN lesions and TD peers or between the most and least affected leg. The results in two studies showed that children with UMN lesions had lower DPS scores of the knee than TD peers (5, 6). Abdin et al. reported significantly lower DPS scores in the more affected leg compared to the less affected leg in 29 children with unilateral cerebral palsy (CP) (7). In contrast, a recent study using 3D motion analysis to assess DPS in 37 children with CP, 11 with myelomeningocele, 19 with arthrogryposis, and 42 TD children found no significant differences in knee DPS among the four groups (8). Damiano found that proprioceptive errors were significantly higher in 32 children with CP than 20 TD peers (9). Wingert and colleagues investigated hip JMS in 38 children with CP and 21 TD peers and concluded that children with CP had a significantly higher onset for detecting JMS than their TD peers (10). None of these studies investigated the feasibility of the proprioceptive assessments.

Based on these results, we developed a child-friendly, portable, sensor-based assessment tool to assess proprioceptive function. This tool, called the Proprioception Measurement Tool (ProMeTo), assesses the three proprioceptive modalities, JMS, JPS, and DPS of the hip, knee, and ankle joints (all without visual input). In addition, to gain insight into the relative contribution of the motor and proprioception components in these proprioception assessments, we also included a test with visual feedback-control of the movements over an avatar.

This study investigated the feasibility, validity, and reliability of the ProMeTo to assess lower limb proprioception in children with UMN lesions. We hypothesised *a priori* that children with UMN lesions have significantly lower proprioceptive function than TD peers (discriminative validity). Furthermore, we expected moderate to good correlations between the three proprioceptive modalities in children with UMN lesions (convergent validity). Finally, we expected moderate to good relative reliability for test-retest and inter-rater reliability, with acceptable errors below 10° for absolute reliability.

## 2 Materials and methods

### 2.1 Study design

We used a cross-sectional observational psychometric study design with repeated assessments to investigate the feasibility, validity, and reliability of the ProMeTo.

### 2.2 Participants

We recruited children and young people with neuromotor impairments due to UMN lesions (e.g., CP, acquired brain injury) from the inpatient and outpatient Swiss Children's Rehab clinic of the University Children's Hospital Zurich. Inclusion criteria were as follows: age 5–19 years, the ability to sit for 30 min with or without back support, and the ability to stand and walk a few steps with or without support. Exclusion criteria were severe visual impairment, lower limb surgery or botulinum toxin injection within the previous six months, inability to communicate pain or discomfort or to follow simple short instructions, and non-compliance. We also recruited TD peers with the following exclusion criteria: any neurological diagnosis, severe visual impairment, developmental coordination disorders, or a diagnosis of attention deficit hyperactivity disorder.

According to the consensus-based standards for selecting health measurement instruments (COSMIN) guidelines, we aimed to recruit 50 children with UMN lesions and 50 TD peers (11). We recorded the Functional Mobility Scale (FMS) score for the distance of 500 m (in community-based settings) as a descriptive measure of the participant's walking ability (12, 13). All children and young people agreed verbally to participate. Parents and adolescents aged 14 years and above also signed an informed consent form. The Cantonal Ethics Committee of Zurich (BASEC-Nr. PB_2021-01373) approved this study, and we followed the good clinical practice guidelines. The study protocol is available on clinicaltrial.gov (trial identifier: NCT05405881).

### 2.3 Test procedure

The measurements took place in a therapy room in the Swiss Children's Rehab or at the practice where the child attends therapy. At the first visit, one assessor used the ProMeTo (test 1) to measure proprioceptive function in the participants. At the second visit, the same assessor repeated the tests (test 2) approximately ten days later. For inter-rater reliability, a sub-sample of the children repeated the assessment after a 5-minute break with a different assessor (assessor A and B). We conducted the repeated measurements for the ProMeTo only within the group of children with UMN lesions.

**Figure 1:**
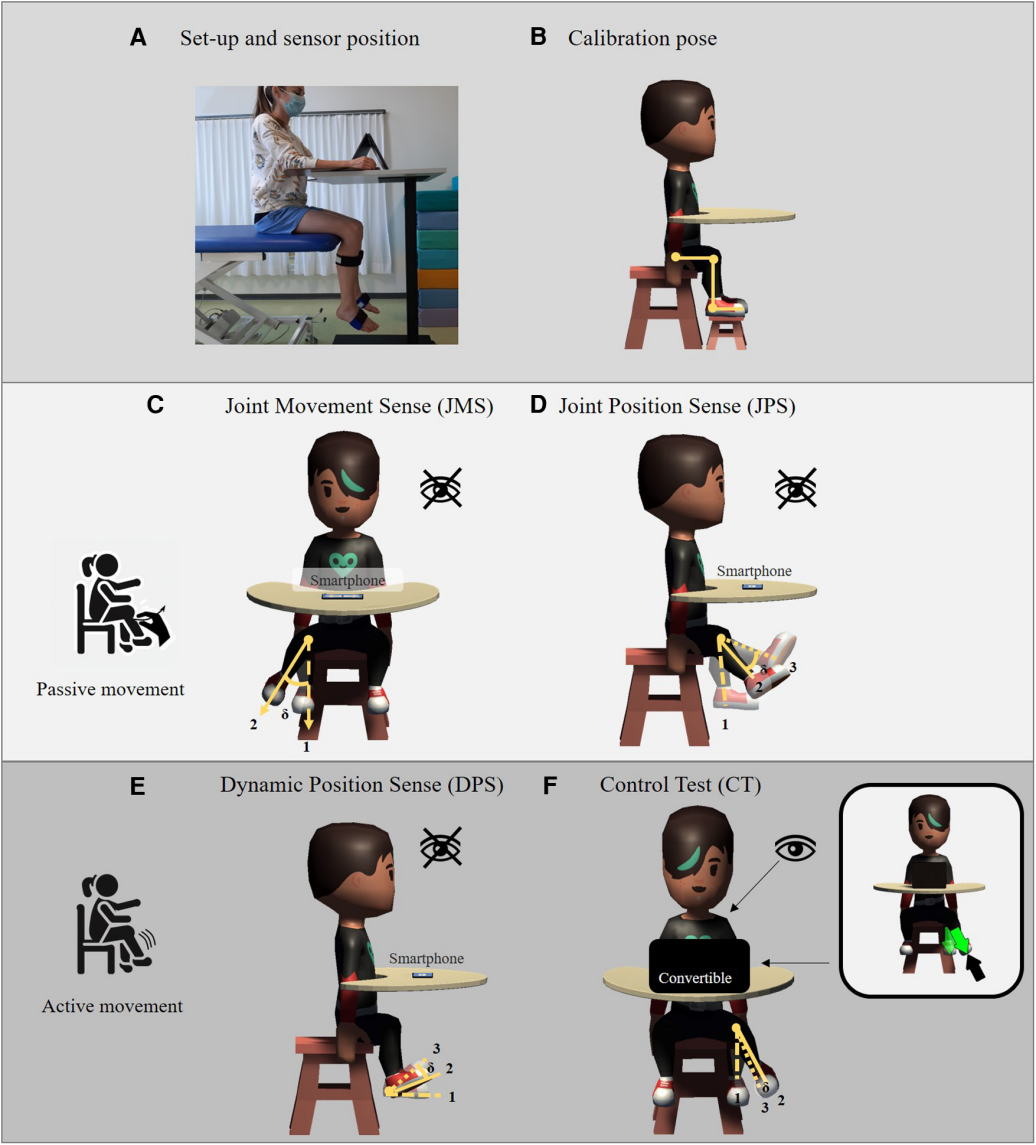
Test setting and modalities of the proprioception measurement tool (ProMeTo). (**A**) Set-up with a U-table preventing the child from seeing his or her legs, and sensor position at the calf and foot with neoprene cuffs; (**B**) Calibration in 90° hip flexion, 90° knee flexion, 90° dorsiflexion, and neutral for rotation and abduction represented in the avatar that guided the assessor through the tests (**C**) Joint movement sense (JMS), the rater moved the leg of the child; (**D**) Joint position sense (JPS), the rater moved the leg of the child; (**E**) Dynamic position sense (DS), the child actively moved the leg toward the criterion position, and (**F**) Control test (CT) where the child had visual feedback showing errors on the convertible notebook. 1 = starting position; 2 = confirmed position of the child; 3 = criterion position; *δ* = difference of the angle between the confirmed and the criterion position.

**Figure 2:**
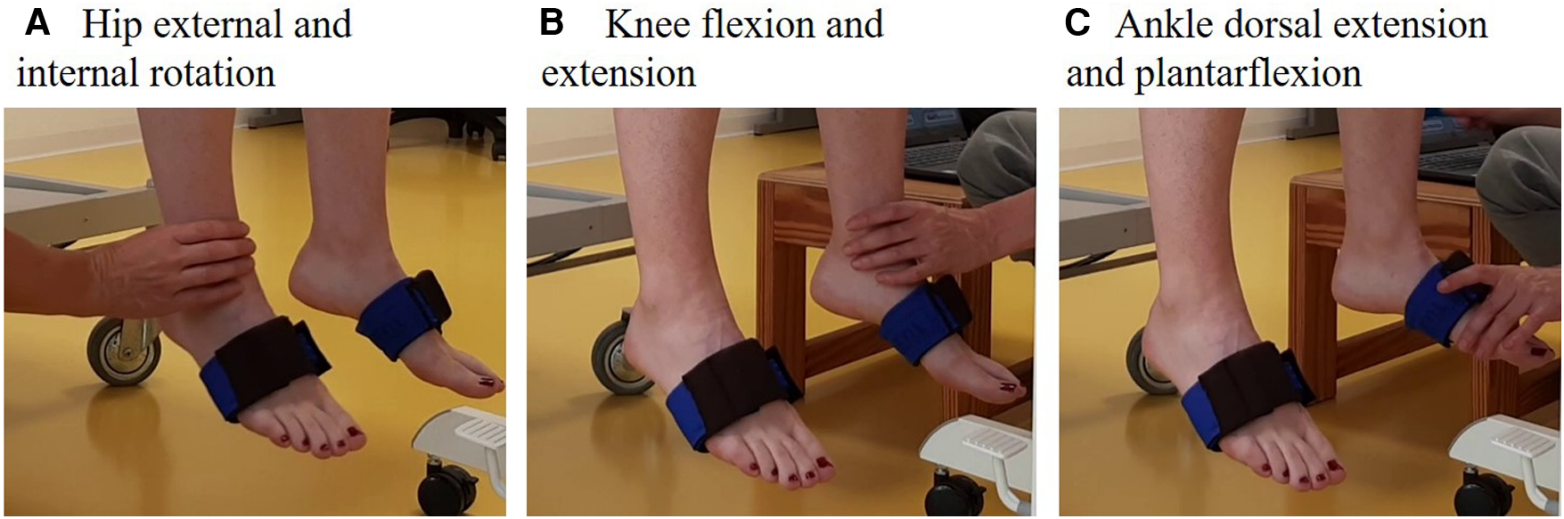
Definition of the grip to hold the leg during the test. (**A**) For hip external and internal rotation, the assessors held the distal lower limb ventral and dorsal with a pincer grip; (**B**) for knee flexion and extension, the assessors held the leg medial and lateral on the malleoli with a pincer grip; and (**C**) for ankle dorsiflexion and plantarflexion, the assessors held the forefoot at the lateral sides with a pincer grip.

We used CE-certified Shimmer inertial measurement units (©Shimmer, Dublin, Ireland) to assess the joint angles. We created an application using the game software Unity (version 2020.4.6f1, Unity Technologies, San Francisco, USA) to guide the assessors through the tests step by step. Figure 1A shows the test setup and sensor application, while Figure 1B shows the calibration position. Back support was provided if needed using a foam pad or assistance from another person. The assessors held the leg distally with predefined grips (Figure 2). The grips were defined to avoid pressure or tension in the applied direction of movement. The assessors used a medium force, comparable to a handshake (around 2.5 N) (14).

The test started with the JMS, followed by the JPS, DPS, and the visual control test. The child played a short game of 25 s between the tests. In the game, the child steered a wizard flexing or extending their knee to collect coins. The game provided fun and allowed the children to have an active break.

For the JMS, the assessor moved the limb at a speed of 5–15°/s in one direction. To avoid moving too fast or slow, the assessor had visual feedback on the convertible notebook to guide the speed. The assessor made one test movement of each joint and used the same terminology as on the digital buttons on the smart phone, to verify the child understood the task. The child was asked to confirm their perceived direction of movement through the digital buttons on a smart phone as fast as possible (Figure 1C). We recorded the percentage of correctly identified directions out of four trials and the angle until detection (°) for each trial.

For the JPS, the assessor moved the limb to a particular position (criterion position) and held this position for three seconds before moving back to the starting position (15). The limb was then slowly (5–15°/s) moved through the whole range by the assessor, and we asked the child to press the digital “Stop” button on the smart phone when they thought their limb had reached the criterion position (Figure 1D). In contrast, for the DPS, we asked the child to actively move their limb to the position that they considered to be the criterion position and press the digital button to confirm (Figure 1E). For each joint, four criterion positions (four trials) for JPS and DPS were assessed; two criterion positions in internal and two in external hip rotation, two positions greater than 90° knee flexion and two less than 90°; and two in ankle dorsiflexion and two in plantarflexion. To prevent selecting a criterion position at the end of the joint range, we assessed the available range of motion for each child by evaluating each joint movement prior to the JPS and DPS tests. The assessor selected the criterion position within the intermediate 80% of the individual range of motion. For JPS, the assessor passively moved the limb twice through the full range and for the DPS, the child actively moved the leg through their active range of motion. For each criterion position, we recorded the difference in degrees (°) between criterion position and the confirmed position (delta) for the JPS and DPS. For both tests, the assessors explained the tasks to the children and let them try out one time to try the test.

The final assessment was the visual control test. To investigate whether the child's motor skills influenced the outcome of the DPS, we used a test with visual feedback as a control test (CT; Figure 1F). The child could see the movement of their limbs on an avatar on the convertible notebook. A target marker on the screen indicated the critical angle that the child had to reach. For each single test, a repetition was possible if the child or the assessor were distracted.

We assessed the feasibility of the ProMeTo by recording testing time, technical issues, and participant feedback on pain, concentration required, and fatigue using a 0–10 visual analogue scale. Verbal feedback and the assessor's assessment using the same scale were also recorded, as well as the child's understanding of instructions (16).

### 2.4 Data and statistical analysis

For the JMS, the percentage value for each joint was calculated based on the correct recognition of the movement direction across four trials, i.e., 100% if the movement direction was correctly recognised in all four trials. We also averaged the angle at which the child recognised the movement direction over the four trials for each joint. For the JPS and DPS, the average delta (criterion position—confirmed position) from the four trials was calculated for each joint.

In addition to the deltas for each joint (hip, knee, and ankle), we also calculated the mean value over the three joints, reflecting the overall proprioceptive error of the leg. We calculated these mean values separately for each modality, child, and the more and less affected leg.

To calculate the proprioceptive component score, the CT was subtracted by the DPS. Statistical analyses were performed using SPSS version 27 (IBM SPSS Statistics, Chicago, IL). Data distribution was evaluated using Shapiro-Wilk tests and visual inspection of the Q-Q plots. For statistical tests, alpha was set to 0.05. Participant characteristics and feasibility data were analysed descriptively.

The results of the first ProMeTo of the children with UMN lesions were used for the statistics for discriminative and convergent validity. For discriminative validity, differences between the two groups were analysed with the Mann-Whitney-*U*-test. For the convergent validity, Spearman correlation coefficients (r_s_) were calculated to quantify the association between the mean values of the three proprioception modalities, and between the proprioceptive component and the JPS. We used the following benchmarks: 0–0.25 (no or little relationship), 0.25–0.50 (fair), 0.50–0.75 (moderate to good), 0.75–1.00 (very good to excellent) (17).

Test-retest reliability and inter-rater reliability were calculated for each joint and the mean value of the three joints per modality using Intraclass Correlation Coefficients (ICCs) and 95% Confidence Intervals (CIs), using the two-way random effect model ICC (2,1) (18). For the interpretation, the following benchmark ICC values were used: lower than 0.25 (poor reliability); 0.26–0.49 (low reliability); 0.50–0.69 (moderate reliability); 0.70–0.89 (high reliability); and higher than 0.90 (very high reliability) (19). The absolute reliability, the standard error of measurement (SEM), and the Smallest Detectable Change (SDC) were calculated with the following formulae: SEM = SD (Standard Deviation) √(1-ICC) and SDC = 1.96x√2xSEM (20). Additionally, we applied Bland-Altman plots to check for systematical bias and the limits of agreements (95% CI) for the test-retest and interrater reliability (21).

## 3 Results

We recruited 51 children with UMN lesions. However, one child did not understand the ProMeTo instructions, and another refused to wear the sensors. Therefore, the data of 49 children (27 girls; 22 boys) with UMN lesions and a mean age of 10.9 years (SD 3.57, range 5–19 years) were available for analysis (Table 1). Fifty TD peers (28 girls; 22 boys) aged 11.5y SD 3.4y served as controls. Levene's test showed that both groups had similar variances for age (*p* = 0.61), height (*p* = 0.31), and weight (*p* = 0.46).

**Table 1:** Characteristics of the children with upper motor neuron lesions and typically developing children.

Variables	Characteristics		Children with UMN lesions (*n* = 49)	Typically developing children (*n* = 50)
Age groups	5 to <10 years		21	19
	10 to <14 years		17	19
	14 to <19 years		11	12
Gender	Girls		27	28
	Boys		22	22
Dominant leg	Right leg		25	35
	Left leg		24	15
Medication	No medication		45	48
	Pain medication		0	0
	Anti-spastic		1	0
	Anti-epileptic		2	0
	Anti-depressive		1	1
	Blood pressure control		0	1
Diagnosis^a^	Cerebral palsy		40	n.a.
	GMFCS	Level I	28	n.a.
		Level II	6	
		Level III	3	
		Level IV	3	
	Stroke		1	n.a.
	Traumatic Brain injury		1	
	Brain tumour		4	
	Others^b^		3	
Functional Mobility Scale: 500 meters	6: Independent on all surfaces		26	50
	5: Independent on level surfaces		14	–
	4: Uses sticks (one or two)		1	–
	3: Uses crutches		0	–
	2: Uses a walker or frame		1	–
	1: Uses wheelchair		7	–
Type of Tone	Mixed tone		18	n.a.
	Spastic		16	
	Ataxia		12	
	Dystonia		3	

UMN, upper motor neuron; GMFCS, gross motor function classification system; n, Numbers; n.a., not applicable.

^a^ Forty-three children had a congenital brain lesion, six an acquired brain lesion.

^b^ Other diagnoses were Joubert syndrome (*n* = 1); Chiari type I and hydrocephalus (*n* = 1); hereditary spastic paresis (*n* = 1).

### 3.1 Feasibility

The three tests lasted, on average, 17 min (SD 7.5 min; range: 9–54 min) for the children with UMN lesions and 15 min (SD 3.1 min; range: 8–22 min) for the TD peers. This difference was not significant (*p* = 0.12). All children with UMN lesions were able to perform the JMS test, but four did not correctly identify the movement in any of the three joints. These four children were also not able complete the JPS and DPS tests due to their low level of proprioception in their legs. Their age ranged between 6 and 12 years, and their diagnoses were CP (two with GMFCS level III and one with GMFCS level IV), and one child with acquired brain lesion due to a stroke.

**Figure 3:**
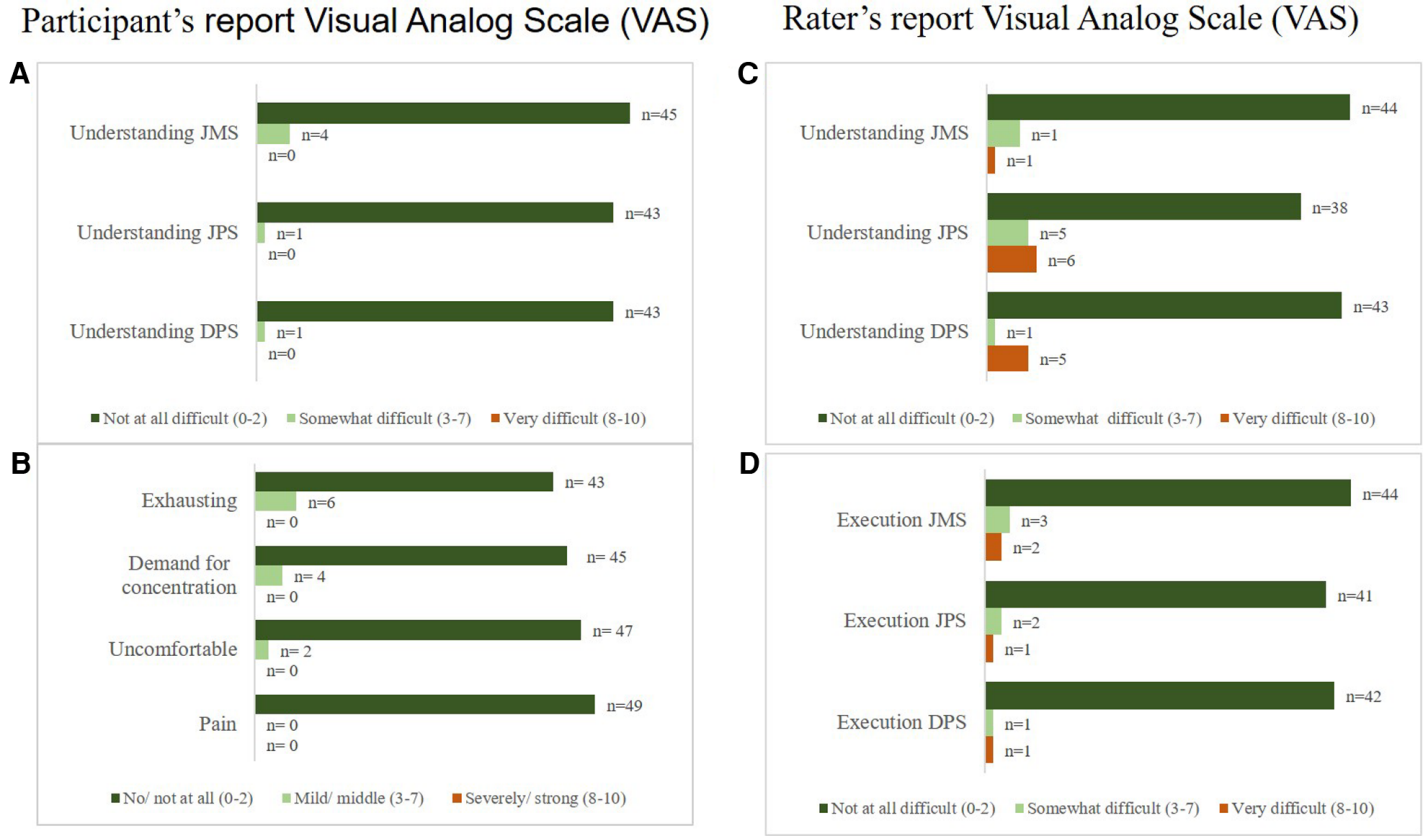
Feasibility results of the children with UMN lesions (*n* = 49). Feasibility results of the children with upper motor neuron lesions reported Visual Analog Scale (VAS) for (**A**) understanding of test modalities and (**B**) exhausting, demand for concentration, uncomfortable, and pain. Further shown is the rater's report (of the first test) for (**C**) interpreting the child's understanding of the test, and (**D**) the execution. Four children (6–12 years) could not perform the JPS and DPS. Their diagnoses were CP with GMFCS levels III (*n* = 2) and IV (*n* = 1), and one child had an acquired brain lesion due to a stroke.

The children with UMN lesions generally understood and accepted the ProMeTo assessments well (Figure 3). Additionally, two children could not perform the DPS of the ankle of their more affected leg. For these two children, the DPS mean scores were calculated based on the hip and knee outcomes.

Overall, executing the tests was relatively easy for the assessors. For a few children who showed voluntary active resistance against the movement, performing the JMS and JPS tests (2 and 3 children, respectively) was particularly difficult. For the TD peers, the tests were generally easy to understand and execute. In addition, the assessors reported the children's high level of comprehension (Supplementary Figure S1).

Verbal feedback from children with UMN lesions indicated varying experiences: some found it fun (*n* = 5), easy to understand (*n* = 2), and enjoyable (*n* = 4), while others found it boring (*n* = 2), slightly tiring (*n* = 2), or uncomfortable due to factors like cold feet or feeling hot under the cuffs with the sensors (*n* = 3). The TD peers generally found it fun (*n* = 5) but noted difficulties in controlling foot movement (*n* = 6) or maintaining leg internal rotation (*n* = 4). Furthermore, they also mentioned that they felt warm under the cuffs (*n* = 2). Only a few technical issues were reported during all the tests, such as occasional loss of connection to the mobile phone (*n* = 4) and slow mobile phone response (*n* = 2).

### 3.2 Hypotheses testing: discriminative and convergent validity

Forty-five children with UMN lesions and 50 TD peers could be included for discriminative and convergent validity testing. In one child with UMN lesions, the JPS data for the more affected leg and the DPS data for the more and less affected leg were missing due to a loss of connection to the sensors; therefore, this analysis included 44 children with UMN lesions.

**Figure 4:**
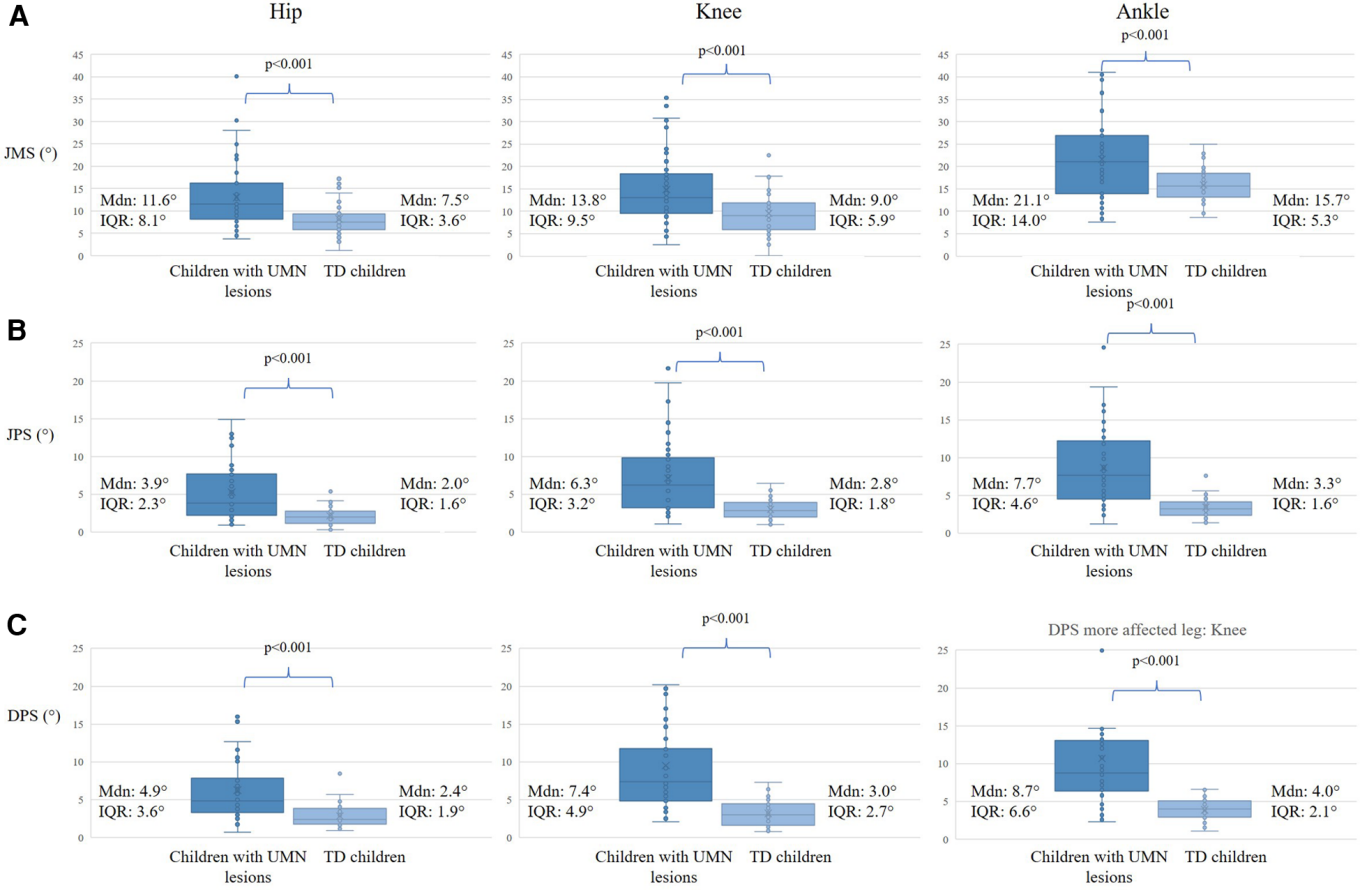
Discriminative validity between children with UMN lesions and typically developing children of the more affected side. The first column shows the hip joint results, the middle column shows the knee joint results, and the right column shows the ankle joint results of the children with UMN lesions and their TD peers. The *p*-value indicates the Mann and Whitney *U*-test with the level of significance for (**A**) Joint Movement Sense (JMS), (**B**) Joint Position Sense (JPS), and (**C**) Dynamic Position Sense (DPS). Further shown are the median values (Mdn) and Interquartile Ranges (IQR). The y-axis represents the test results in degrees (°).

The children with UMN lesions had significantly higher test values (i.e., lower proprioceptive function) than their TD peers for all tests and all joints (Figure 4, more affected leg; Supplementary Figure S2, less affected leg).

**Figure 5:**
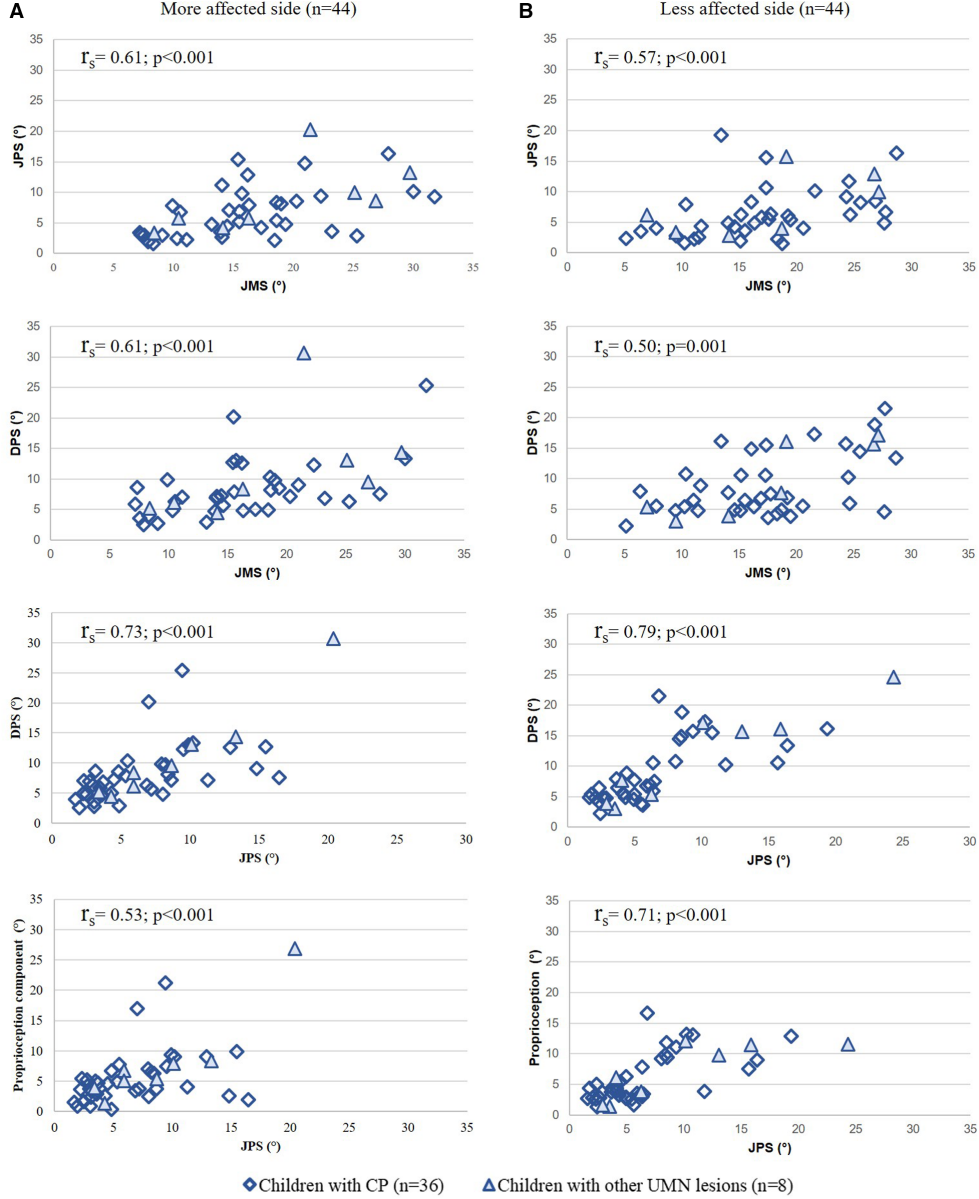
Convergent validity results: correlation between the mean values of the modalities and the proprioception component of the (**A**) more affected side and (**B**) less affected side. Spearman correlations (r_s_) and *p*-values between all modalities. Y- and x-axis represent the test results in degrees (°).

Correlation coefficients between the mean values of JMS and JPS, and between JMS and DPS were moderate to good for both the more (r_s_ 0.53–0.73; *p* < 0.001) and less affected side (r_s_ 0.50–0.79; *p* < 0.001; Figure 5). The correlation between the JPS and DPS for both the more and less affected sides was good to excellent. Also, the relationship between the proprioceptive component (DPS subtracted by the CT) and the JPS was moderate to good for both the more and less affected sides (Figure 5).

### 3.3 Test-retest and inter-rater reliability

Forty-six children completed the ProMeTo twice (the first test and the second test). Four children did not correctly identify the direction of joint movement in both tests. Therefore, we included 42 children in the test-retest reliability analyses for the JMS. For the JPS and DPS, two children's records of the second test were missing (*n* = 1 with CP, classified GMFC level IV, *n* = 1 status after brain tumour); therefore, we included 40 children in these analyses.

The relative reliability was high for the more and less affected side, except for the JMS for the ankle on the less affected side, which was moderate (Table 2). Absolute reliability, expressed by SDC, lay below 10°, except for the JMS (Table 2). The Bland-Altman plots represent the bias for the more affected leg for JMS of 0.6° (95% CI −6.7° to 7.9°), and for the JPS 0.3° (95% CI −2.9° to 3.5°), and for the DPS −0.1° (95% CI −3.7° to 3.6°), and comparable results for the agreement of the less affected leg (Supplementary Figure S3).

**Table 2:** Test-retest reliability of the proprioception modalities assessed in children with UMN lesions.

		More affected leg					Less affected leg				
Modality, values	(*n*)	Test 1: Mean ± SD (°)	Test 2: Mean ± SD (°)	ICC (95% CI)	SEM (°)	SDC (°)	Test 1: Mean ± SD (°)	Test 2: Mean ± SD (°)	ICC (95% CI)	SEM (°)	SDC (°)
JMS, hip	42	13.3 ± 7.4	13.1 ± 9.2	0.90 (0.83–0.95)*	2.6	7.3	14.0 ± 7.0	13.8 ± 9.0	0.77 (0.60–0.87)*	3.8	10.7
JMS, knee	42	15.0 ± 8.1	15.0 ± 8.9	0.74 (0.56–0.85)*	4.3	12.0	17.4 ± 10.5	16.2 ± 11.0	0.76 (0.60–0.87)*	5.2	14.5
JMS, ankle	42	22.0 ± 9.1	20.5 ± 8.5	0.76 (0.59–0.86)*	4.2	11.9	23.6 ± 8.8	22.9 ± 9.1	0.66 (0.44–0.80)*	5.2	14.4
JMS, mean	42	16.8 ± 6.7	16.2 ± 7.6	0.87 (0.78–0.93)*	2.6	7.2	18.3 ± 7.6	17.6 ± 8.5	0.79 (0.64–0.88)*	3.7	10.1
JPS, hip	40	5.4 ± 4.0	4.9 ± 4.1	0.89 (0.80–0.94)*	1.3	3.6	5.3 ± 3.5	4.4 ± 3.4	0.83 (0.69–0.90)*	1.3	3.6
JPS, knee	40	7.1 ± 5.0	6.8 ± 5.3	0.90 (0.82–0.95)*	1.6	4.4	7.1 ± 6.9	6.9 ± 6.3	0.94 (0.88–0.97)*	1.5	4.2
JPS, ankle	40	8.8 ± 5.7	7.8 ± 4.6	0.86 (0.76–0.93)*	1.9	5.4	9.3 ± 6.5	8.8 ± 5.9	0.88 (0.78–0.93)*	2.0	5.5
JPS, mean	40	7.1 ± 4.5	6.5 ± 4.2	0.93 (0.86–0.96)*	1.7	4.8	7.2 ± 5.2	6.7 ± 4.6	0.94 (0.89–0.98)*	1.1	3.1
DPS, hip	40	6.4 ± 4.0)	6.5 ± 4.7	0.79 (0.64–0.88)*	2.2	6.0	7.0 ± 5.0	6.5 ± 4.5	0.82 (0.70–0.90)*	2.3	6.4
DPS, knee	40	10.0 ± 7.6	9.2 ± 6.8	0.90 (0.82–0.94)*	2.3	6.4	9.9 ± 7.4	9.3 ± 6.3	0.86 (0.75–0.92)*	2.6	7.1
DPS, ankle	39/40	10.9 ± 8.0	11.3 ± 8.1	0.93 (0.87–0.96)*	2.1	6.0	11.9 ± 8.2	11.2 ± 8.3	0.91 (0.83–0.5)*	2.2	6.2
DPS, mean	40	8.7 ± 5.8	8.9 ± 5.6	0.95 (0.91–0.97)*	1.3	3.6	9.6 ± 5.7	8.9 ± 5.4	0.92 (0.86–0.96)*	1.6	4.3

SD, standard deviation; CI, confidence interval; ICC, intraclass correlation coefficient; SEM, standard error of measurement; SDC, smallest detectable change.

* *p* < 0.001.

The data from sixteen children were used for the inter-rater reliability analysis. Relative reliability was high for all modalities (Supplementary Table S1). Absolute reliability, expressed by SDC, was below 10°, except for the JMS (Supplementary Table S1). The Bland-Altman plots represent the bias for the more affected leg for JMS of −3.0° (95% CI −11.3° to 5.4°), and for the JPS 0.1° (95% CI −2.8° to 3.0°), and the DPS −0.4° (95% CI −3.3° to 2.6°), and comparable results for the agreement of the less affected leg (Supplementary Figure S3).

## 4 Discussion

We determined the feasibility, validity, and reliability of three modalities of the lower limb in children with UMN lesions. Our main results are: (i) the ProMeTo is feasible to assess children with UMN lesions, (ii) the tests showed high discriminative and acceptable convergent validity, and (iii) the test-retest reliability was moderate to high, with the SDC generally below 10° for the more affected leg, except for the JMS.

The children's acceptance of and compliance with the test was appropriate for all modalities. Overall, it was easy for the assessors to apply the assessments to the children. An advantage of the ProMeTo is that it is portable, so that we were able to test the children in our clinic, at their homes, at the location where they receive therapy, or at their schools. The sensors make it possible to test three joints of the lower limbs, each in two directions of movement, in a relatively short period. The short breaks when they played the game between the test modalities appeared to help keep the children's attention and motivation high. Although some studies have assessed proprioception in children with UMN lesions, we cannot compare our feasibility results because they did not investigate feasibility (5, 6, 9, 10). The ProMeTo required children to have a certain level of upper limb function to be able to press the digital button. However, given children's familiarity with digital gadgets today, our feasibility was remarkably high.

Children with UMN lesions had lower proprioceptive function than their TD peers assessed with the ProMeTo. This result is in line with previous studies assessing DPS of the knee (5, 6) and the hip (9, 10). However, in the study by Bartonek and colleagues, no significant difference in the DPS of the knee was observed between children with motor impairments and TD children (8). A recent study investigated the threshold of the ankle angle perceived by children with CP and TD as a measure reflecting JPS (22). Children with CP had a statistically higher threshold than TD children, reflecting lower JPS acuity. Despite the statistical difference, they found a huge overlap in ankle JPS threshold values between the children with CP and the TD children. Only one of 16 children with CP exceeded the normal distribution obtained in the TD children (overlap of children of 94%). Our study's overlap was 48% (21 of 44 children). As the participants' characteristics in our studies were comparable, the findings might indicate that our measurement protocol for JPS for the ankle discriminated better between children with a UMN lesion and TD children.

It is noteworthy that all these studies employed specifically defined criterion angles, which may have introduced a potential learning effect influencing the test results. In our study, the criterion positions were not predefined, thereby preventing the child from estimating the angle based on previous trials. Nevertheless, the different positions of criterion positions could also negatively influence the test's reproduction and, therefore, our reliability results. By avoiding extreme positions in the end range of motion, we tried to control for the influence of joint position on proprioceptive acuity.

The three modalities showed a moderate to good relationship with each other. We found the highest relationship between the JPS and the DPS. No previous study has investigated the convergent validity of different proprioceptive modalities in children with UMN lesions. Using the proprioceptive component score, we corrected the DPS by subtracting the result of the visual control test (i.e., the motor component). The good relationship between the proprioceptive scores and the JPS confirms the convergent validity of the JPS and DPS. Further, we conclude that proprioception influenced the retrieval of the criterion angle and that the limitation in the DPS was not primarily due to the motor limitation.

The relative reliability of the ProMeTo was moderate to high for individual joints, and the values averaged over all three joints for each of the three modalities.

To date, no study has investigated the reliability of proprioceptive assessment in children with UMN lesions. To interpret the absolute reliability values, we need to investigate which changes can be induced by therapeutic intervention and establish which differences are clinically meaningful for the children. For example, Ko et al. investigated the efficacy of a 3-week whole body vibration intervention compared to a control intervention in 24 children with CP, 12 in each group. They found a significantly higher improvement in ankle DPS of the dominant leg in the intervention group (mean improved DPS was about 5.9°). In our study, we found a SEM value of 2.2° for the ankle joint of the less affected leg. Therefore, we assume that our DPS assessment might be sensitive enough to detect therapy-induced changes (23).

Further, we need to establish which differences (errors) impact these children's motor function, quality of movement, and movement performance. In our study, SDC was under 10° for JPS and DPS but only under 15° for JMS. The JMS's absolute reliability is, therefore, rather low (large difference), and possible changes within an individual can probably not be detected. Thus, our hypotheses were confirmed for JPS and DPS but not for JMS, possibly due to variations in children's results influenced by longer motion identification distances.

### 4.1 Methodological considerations

The Shimmer sensors are anti-gravity sensors and had an effect on the choice of direction of movement, particularly for the hip (rotation instead of flexion and extension). Overall, the methodological quality of this psychometric study is “fair” according to the COSMIN guidelines due to the moderate sample size (11). We assessed 41 children twice for test-retest reliability and only 16 for intra-rater reliability. Therefore, the generalisability of these reliability results needs to be cautiously interpreted. Another limitation is that most children with CP (70%) were classified as GMFCS I, meaning their motor skills are generally good. Despite this high proportion of children with good motor skills in the UMN lesion group, the difference in proprioception function between the two groups was statistically significant.

As proprioception is multimodal and uses tactile information, our results can partly be influenced by how the assessor held and moved the limb (24) or the selected position of the criterion positions. Considering the force of the grip and the position, we standardised this as much as possible to avoid the tactile input, which would indicate the direction and range of joint movement. Still, we cannot exclude the fact that the children used tactile input in their strategy to perform the test. Considering the criterion positions, one suggestion for improvement could be to standardize the testing angle within a predefined intermediate range of motion, more precisely on a specific angle, to account for factors affecting proprioceptive acuities, such as receptor sensitivity or ligament and muscle tension. Such a protocol, however, would take more time because the therapist needs to position the joint at the exact angles, which might affect the feasibility and compliance of the child. In our study, the criterion angle differed between participants and between the repeated tests. Despite these differences' criterion angles, the reliability was still high. Further, we only reported the results for each joint and each leg and not for each set criterion angle, as we randomly selected these for each participant.

As not all the data were normally distributed, we applied nonparametric tests. Even though we calculated ICCs for relative reliability we also applied the ICC on the log-transformed data and the results were of the same level of interpretation as the ICCs of the original data.

## 5 Conclusion

The ProMeTo offers a feasible, valid, and reliable tool to assess lower limb proprioception in children with UMN lesions. The portable equipment allows the assessment of children directly at their therapy place.

## Data Availability

The raw data supporting the conclusions of this article will be made available by the authors, without undue reservation.

## Supplementary material

Supplementary Figure 1 Feasibility results of the typically developing peers (*n* = 50). Feasibility results of the typically developing peers reported Visual. Analog Scale (VAS) for (**A**) understanding of the test modalities, (**B**) exhausting, demand for concentration, uncomfortable, and pain. Further shown is the rater's report (of the first test) for (**C**) interpreting the understanding of the test of the child, and (**D**) the execution.

Supplementary Figure 2 Discriminative validity between children with UMN lesions and typically developing children of the less affected side. The first column shows the hip joint results, the middle column shows the knee joint results, and the right column shows the ankle joint results of the children with UMN lesions and their TD peers. The *p*-value indicates the Mann and Whitney U-test with the level of significance for (**A**) Joint Movement Sense (JMS), (**B**) Joint Position Sense (JPS), and (**C**) Dynamic Position Sense (DPS), and the median values (Mdn) and Interquartile Ranges (IQR). The y-axis represents the test results in degrees (°).

Supplementary Figure 3 Bland-Altman-Plots, with the bias, and the Lower Limit (LL) and Upper Limit (UL) of Agreement. Test-retest and interrater agreement of the (**A**) Joint Movement Sense, (**B**) Joint Position Sense, and (**C**) Dynamic Position sense mean values.
