# Supplementary figures and images for: Assessing proprioception in children with upper motor neuron lesions: feasibility, validity, and reliability of the proprioception measurement tool
Source: Front Rehabil Sci. 2024 Aug 9;5:1373793. doi: 10.3389/fresc.2024.1373793 (PMC11341540; doi:10.3389/fresc.2024.1373793)

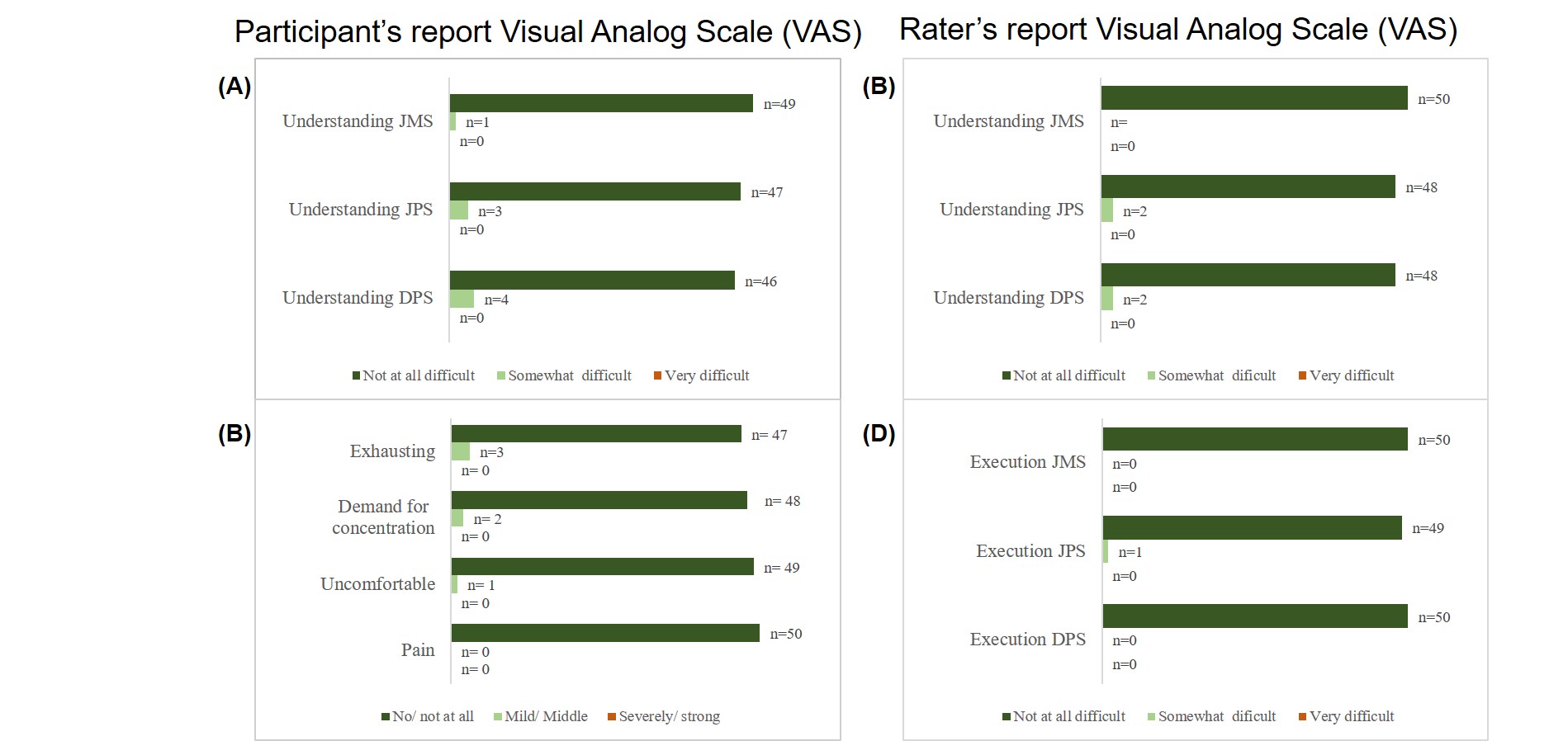

Supplement: Supplementary Figure 1 — Feasibility results of the typically developing peers (n = 50). Feasibility results of the typically developing peers reported Visual. Analog Scale (VAS) for (A) understanding of the test modalities, (B) exhausting, demand for concentration, uncomfortable, and pain. Further shown is the rater's report (of the first test) for (C) interpreting the understanding of the test of the child, and (D) the execution. [file Image1.jpeg]

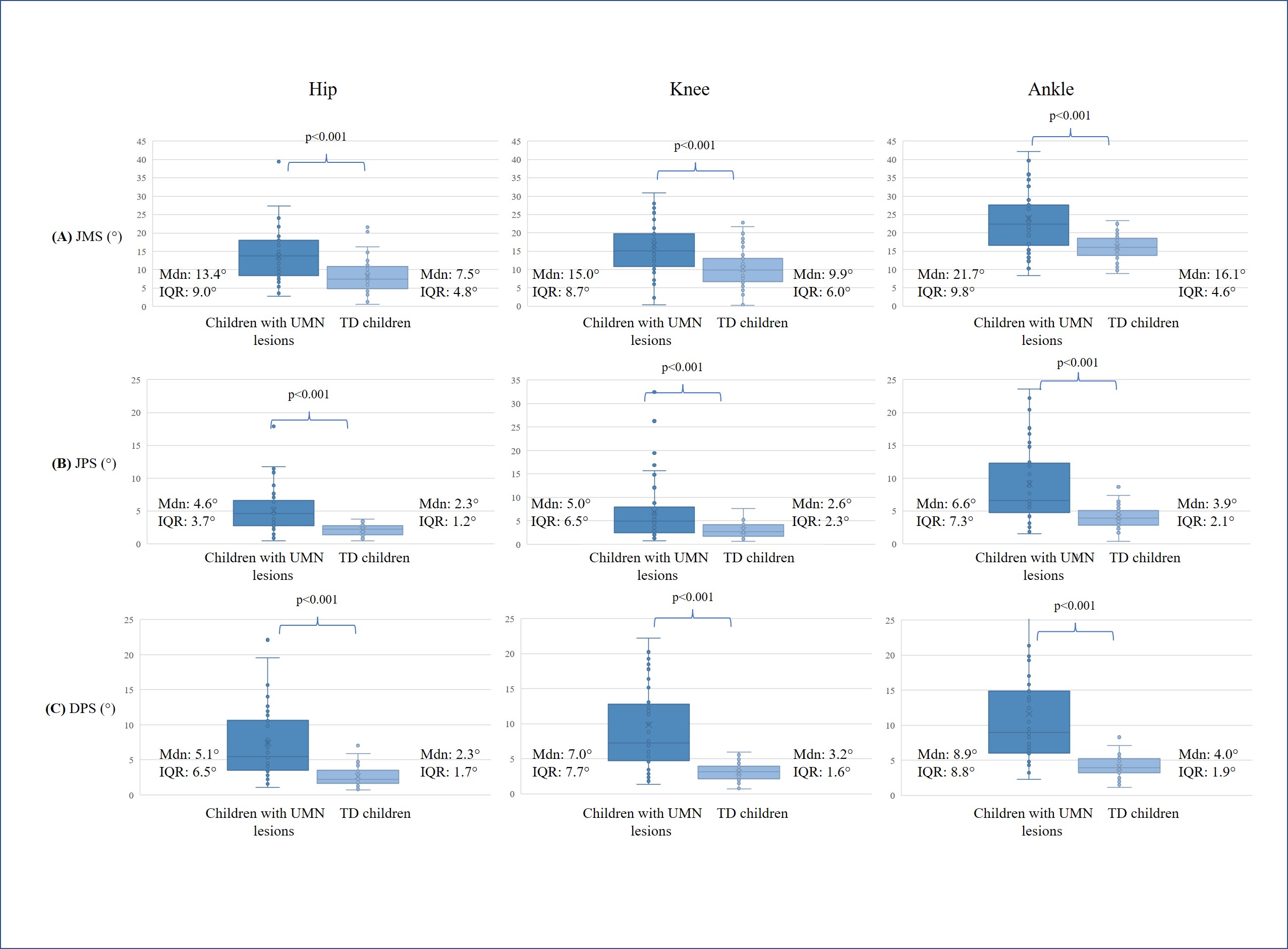

Supplement: Supplementary Figure 2 — Discriminative validity between children with UMN lesions and typically developing children of the less affected side. The first column shows the hip joint results, the middle column shows the knee joint results, and the right column shows the ankle joint results of the children with UMN lesions and their TD peers. The p-value indicates the Mann and Whitney U-test with the level of significance for (A) Joint Movement Sense (JMS), (B) Joint Position Sense (JPS), and (C) Dynamic Position Sense (DPS), and the median values (Mdn) and Interquartile Ranges (IQR). The y-axis represents the test results in degrees (°). [file Image2.jpeg]

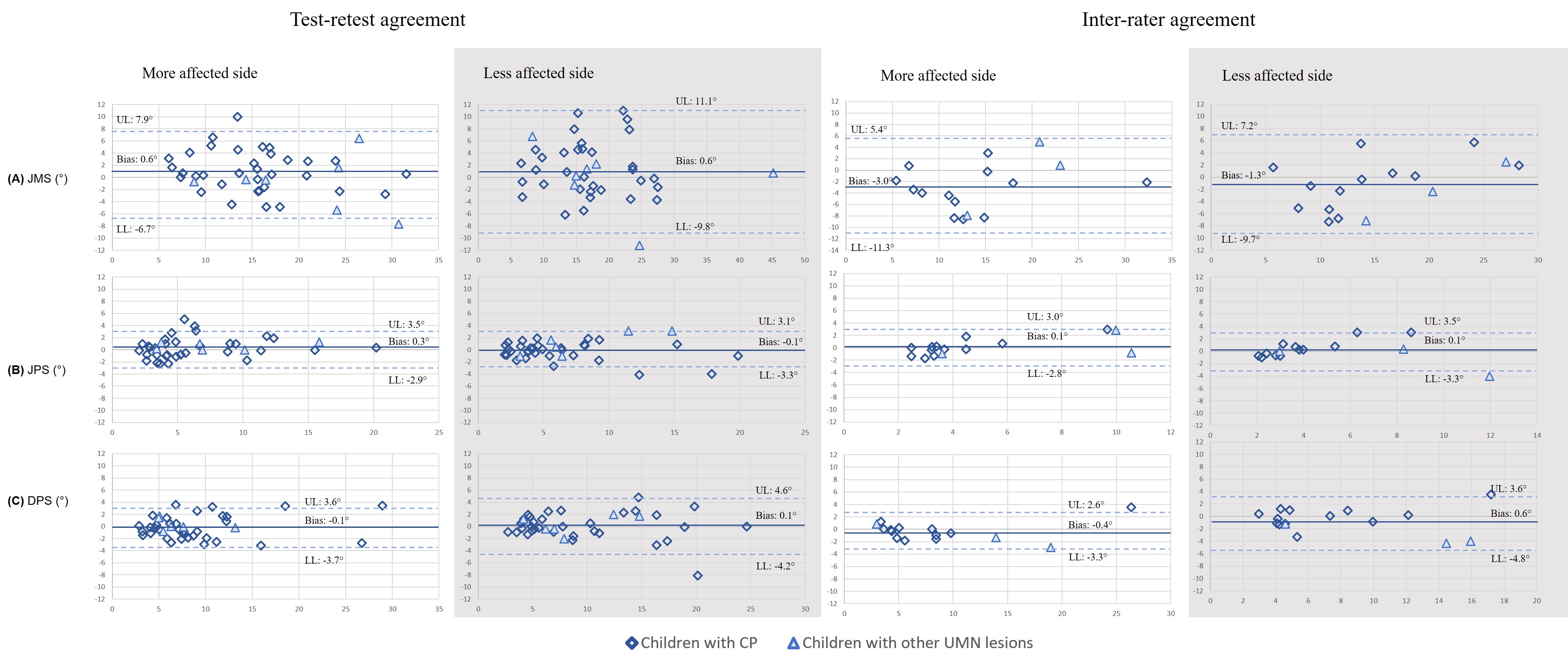

Supplement: Supplementary Figure 3 — Bland-Altman-Plots, with the bias, and the Lower Limit (LL) and Upper Limit (UL) of Agreement. Test-retest and interrater agreement of the (A) Joint Movement Sense, (B) Joint Position Sense, and (C) Dynamic Position sense mean values. [file Image3.jpg]
