# Supplementary material for: Assessing proprioception in children with upper motor neuron lesions: feasibility, validity, and reliability of the proprioception measurement tool
Source: Front Rehabil Sci. 2024 Aug 9;5:1373793. doi: 10.3389/fresc.2024.1373793 (PMC11341540; doi:10.3389/fresc.2024.1373793)
Supplement: Supplementary file 4 [file Table1.docx]

| Supplementary Table S1. Inter-rater reliability of the proprioception measures assessed in children with UMN lesions. | | | | | | | | | | | |
| --- | --- | --- | --- | --- | --- | --- | --- | --- | --- | --- | --- |
|  | | **More affected leg** | | | | | **Less affected leg** | | | | |
| Modality, values | n | Rater A: Mean ±SD (°) | Rater B: Mean ±SD (°) | ICC (95% CI) | SEM (°) | SDC (°) | Rater A: Mean ±SD (°) | Rater B: Mean ±SD (°) | ICC (95% CI) | SEM (°) | SDC (°) |
| JMS, hip | 16 | 10.3±6.7 | 14.0±6.4 | 0.70 (0.23-0.89)** | 3.7 | 10.2 | 10.9±6.7 | 10.7±4.1 | 0.65 (0.22-0.86)* | 3.3 | 9.0 |
| JMS, knee | 16 | 11.1±9.7 | 15.3±7.4 | 0.70 (0.21-0.89)** | 4.8 | 13.4 | 11.5±8.7 | 14.4±7.8 | 0.77 (0.43-0.91)** | 4.0 | 11.0 |
| JMS, ankle | 16 | 16.7±9.3 | 18.5±11.1 | 0.75 (0.43-0.90)** | 4.9 | 13.5 | 21.7±11.6 | 22.5±8.0 | 0.73 (0.38-0.89)** | 5.1 | 14.2 |
| JMS, mean | 16 | 12.7±7.7 | 15.2±6.9 | 0.77 (0.35-0.92)** | 3.4 | 9.5 | 14.7±8.0 | 16.0±5.7 | 0.83 (0.60-0.94)** | 2.9 | 7.8 |
| JPS, hip | 15 | 3.7±2.7 | 3.9±3.0 | 0.80 (0.50-0.93)** | 1.2 | 3.5 | 3.2±2.8 | 3.4±3.2 | 0.93 (0.81-0.98)** | 0.8 | 2.1 |
| JPS, knee | 15 | 4.7±3.4 | 4.1±2.4 | 0.77 (0.45-0.92)** | 1.4 | 3.9 | 4.4±3.0 | 4.5±3.2 | 0.82 (0.54-0.93)** | 1.3 | 3.6 |
| JPS, ankle | 15 | 6.4±4.5 | 6.6±3.9 | 0.76 (0.42-0.91)** | 2.0 | 5.6 | 6.6±4.5 | 7.2±5.3 | 0.88 (0.68-0.96)** | 1.7 | 4.7 |
| JPS, mean | 15 | 4.9±3.3 | 4.8±2.4 | 0.88 (0.69-0.96)** | 1.0 | 2.7 | 4.8±2.8 | 4.6±3.1 | 0.85 (0.60-0.95)** | 1.2 | 3.3 |
| DPS, hip | 15 | 7.3±6.3 | 7.7±5.5 | 0.89 (0.72-0.96)** | 1.9 | 5.3 | 6.4±5.3 | 7.3±6.1 | 0.92 (0.77-0.97)** | 1.6 | 4.4 |
| DPS, knee | 15 | 8.7±8.1 | 8.7±6.8 | 0.93 (0.81-0.98)** | 1.9 | 5.4 | 8.5±6.2 | 8.7±6.0 | 0.81 (0.52-0.93)** | 2.6 | 7.3 |
| DPS, ankle | 14/15 | 9.8±8.8 | 10.4±9.1 | 0.97 (0.91-0.99)** | 1.5 | 4.2 | 8.2±4.8 | 8.9±4.6 | 0.81 (0.53-0.93)** | 2.0 | 5.6 |
| DPS, mean | 15 | 8.5±6.7 | 8.7±6.5 | 0.97 (0.93-0.99)** | 1.1 | 3.1 | 7.7±4.8 | 8.3±5.0 | 0.91 (0.76-0.98)** | 1.5 | 4.0 |
| Abbreviations: SD – Standard Deviation; CI – Confidence Interval; ICC – Intraclass Correlation Coefficient; SEM – Standard Error of Measurement; SDC – Smallest Detectable Change  **p<0.001; *p<0.01 | | | | | | | | | | | |
|  | | | | | | | | | | | |
